# Effect of six-week short-duration deep breathing on young adults with chronic ankle instability-a pilot randomized control trial

**DOI:** 10.1186/s13102-023-00758-5

**Published:** 2023-11-15

**Authors:** Vinodhkumar Ramalingam, Soon Keng Cheong, Poh Foong Lee

**Affiliations:** 1 Saveetha College of Physiotherapy, Saveetha Institute of Medical and Technical Sciences, Saveetha University, Chennai, India; 2 Faculty of Health and Life Sciences, INTI International University, Nilai, Malaysia; 3 Faculty of Medicine and Health Sciences, University Tunku Abdul Rahman, Bandar Sungai Long, Kajang, Selangor Malaysia; 4 Lee Kong Chian Faculty of Engineering & Science, University Tunku Abdul Rahman, Bandar Sungai Long, 43000 Kajang, Selangor Malaysia

**Keywords:** Ankle sprain, Attention, Chronic Pain, Conventional physiotherapy, Short duration deep breathing

## Abstract

**Background:**

Chronic ankle instability (CAI) is the most common injury in youth sports, which leads to psychological stress from doubting their performance. Cost effective and easy to access tool to reduce the stress among this target group are desired. Therefore, the purpose of this study was to investigate the effect of adding on intervention with short-duration deep breathing (SDDB) alongside with conventional physiotherapy (CP) among young adults with chronic ankle instability (CAI).

**Methods:**

Total of 30 CAI participants attended physiotherapy, who were randomly assigned into control and experimental groups. The participants in the experimental group received combined intervention (SDDB + CP), and the control group received CP for 6 weeks. The effectiveness of interventions was assessed at 3 intervals with a battery of questionnaires (Visual Analog Score, Cumberland Ankle Instability Tool, Mindful Attention Awareness Scale, and Oxford Happiness Questionnaire) at the end of week 3, week 6, and week 12 as follow-up. A two-way repeated measures of ANOVA was applied to report the statistical significance at *p* < 0.05.

**Results:**

The results showed a better improvement in pain, balance, happiness, and mindfulness attention among participants in the experimental group, with a significant improvement in mindful attention over the time point as compared to the control group.

**Conclusion:**

The findings provide insight into incorporating SDDB additions to the existing CP for better CAI management. Breathing techniques that improve attention and happiness play a vital role in CAI, which recommends the biopsychosocial approach in chronic injury rehabilitation.

**Trial registration:**

Current Controlled Trials using Clinical Trials Registry under ID number NCT04812158 retrospectively registered on 23/03/2021.

**Supplementary Information:**

The online version contains supplementary material available at 10.1186/s13102-023-00758-5.

## Introduction

Chronic ankle instability (CAI) is a phenomenon of repeated instability of the lateral ligament of the ankle, leading to repeated sprains of the affected ankle. The unstable condition of the lateral ligament of the ankle is mainly caused by an individual sprain in various accidental situations. About 40% of ankle injuries are found to cause repeated ankle sprains [1] that alter the biomechanics of gait [2]. The most common sprain occurs during landing, such as landing downstairs and landing in various ball games [3], and is specific to landing on one leg [4], which causes non-contact lower limb injuries [5]. In that the ankle sprains present with pain and swelling around the ankle, loss of muscle strength, repetitive sprains, and functional ankle instability [6]. CAI is a functional deficit in ankle movement that affects the quality of life of an affected individual, especially during sports [7], and if left untreated, can cause repetitive ankle injury and potentially severe the movement impairments [8]. Additionally, this repetitive ankle ligament injury in the CAI would have an effect on central nervous system excitability functions [9] by altering motor evoked potential and cortical activation in a way that results in deficits in the motor thresholds of the brain [10] and causes neurocognitive deficits [11]. These changes disturb the normal balance and coordination because of the alterations in the mechanoreceptors of the peripheral joint that cause them to lose their position sense in CAI individuals [12]. Prior CAI reports also showed a significant linear relationship between ankle stability and attention [13]. The target sample, which focused on male college students with CAI exhibited deficiencies in neurocognitive performance that can affect posture stability, hence resulting in recurrence of repetitive ankle sprains [11].

According to the updated model of CAI (2019), the main reason for patients to seek rehabilitation programmes for CAI is persistent pain [14], which is caused by cortical changes in the brain following repeated ankle ligament injuries [10] that affect the patient's balance following peripheral and central influences in controlling ankle movements [9]. In health care settings, the visual analogue scale (VAS) is primarily used to quantify pain subjectively [15], while the ankle's functional balance is assessed using the Cumberland Ankle Instability Tool (CAIT), with a point total of less than or equal to 27 [16]. Furthermore, CAI rehabilitation, as per existing studies, focuses primarily on repairing injured athletes' peripheral responses but not on psychological assessments of their attention that impact the central nervous system [17, 18]. As evidenced, focused mindfulness or deep breathing techniques improve attention [19], reduce stress [20], and well-being [21], which in turn improves happiness [22, 23]. Besides that, continuous deep breathing training improved participants' brain function by reducing the respiratory flow rate and improving the cerebral oxygen supply [24]. Further, deep breathing was found to be the most effective method for stress management and competition preparation among collegiate athletes [25]. Whereas the 15-item Mindful Attention Awareness Scale (MAAS) questionnaire [26, 27] and the 29-item Oxford Happiness Questionnaire (OHQ) [28] are used to assess attention and happiness, respectively.

In general, the physiological effects of deep breathing stimulate the wandering nerve "vagal tone" in accordance with polyvagal theory by increasing parasympathetic activity for well-being [29]. As proposed, breathing techniques are contemplative activities that produce structural changes in cognitive neuroscience research and the well-being of practitioners [30]. Deep breathing is a non-pharmacological pain management therapy [31] that improves localised tissue circulation and shifts the focus away from the pain that injured athletes experience [32]. Guided or focused deep breathing acts as a mindful meditation technique [33] that engages the diaphragm [34] by stimulating the vagal nerve [29] to control pain perception [35] and induces a change in sympathetic nervous system activity by lowering stress hormone levels [20]. Also, deep breathing has been proven to be helpful as an effective self-management technique for patients with chronic musculoskeletal pain in terms of reducing disability and improving quality of life and subjective well-being [36]. Furthermore, recent research on the impact of short-duration breathing techniques found that 3 min of breathing exercise had a better physiological effect [37] in acute pain [38], chronic ankle pain [39], fatigue [40], than 5, 7 and 9 min of video-assisted deep breathing [41]. Thus far, no study has been reported on the effectiveness of short-duration deep breathing (SDDB) in CAI to address the neurocognitive deficits such as attention. Since the duration of practise has no effect on the participants' quality of life or anxiety [42], the present study introduced 3 min video assisted short-duration deep breathing to examine the physiological effects in CAI participants. The assessment and deep breathing training to address the central influence of CAI rehabilitation is scanty, and hence there is a need to study the individual response before and after the intervention. A verified method of SDDB was adopted in this study [39, 41, 43]. This is the invention of the same team who modified the video to 3 min and integrated it into an App to benefit the wider community.

In this study, we aimed to investigate the combined effects of a guided 3-min SDDB technique and conventional physiotherapy (CP) for a period of 6 weeks over the control group that received only CP for 6 weeks on male athletes with CAI. The outcome measures of the interventions were assessed subjectively using the VAS, CAIT, MAAS and OHQ. We predicted that a 6-week SDDB programme would significantly reduce subjective pain and improve ankle stability, attention, and happiness in CAI.

## Materials and methods

### Participants

This pilot, randomized, and parallel-group (ratio of 1:1) study was conducted by recruiting participants to control group (CG) and experimental group (EG). The study protocols were approved by the local university's research ethical review committee to meet the compliance with the Helsinki Declaration. Furthermore, the study protocol was registered under the clinical trials registry (NCT04812158). Written informed consent was acknowledged by all the participants before the assessment and intervention.

### Procedures

**Fig. 1:**
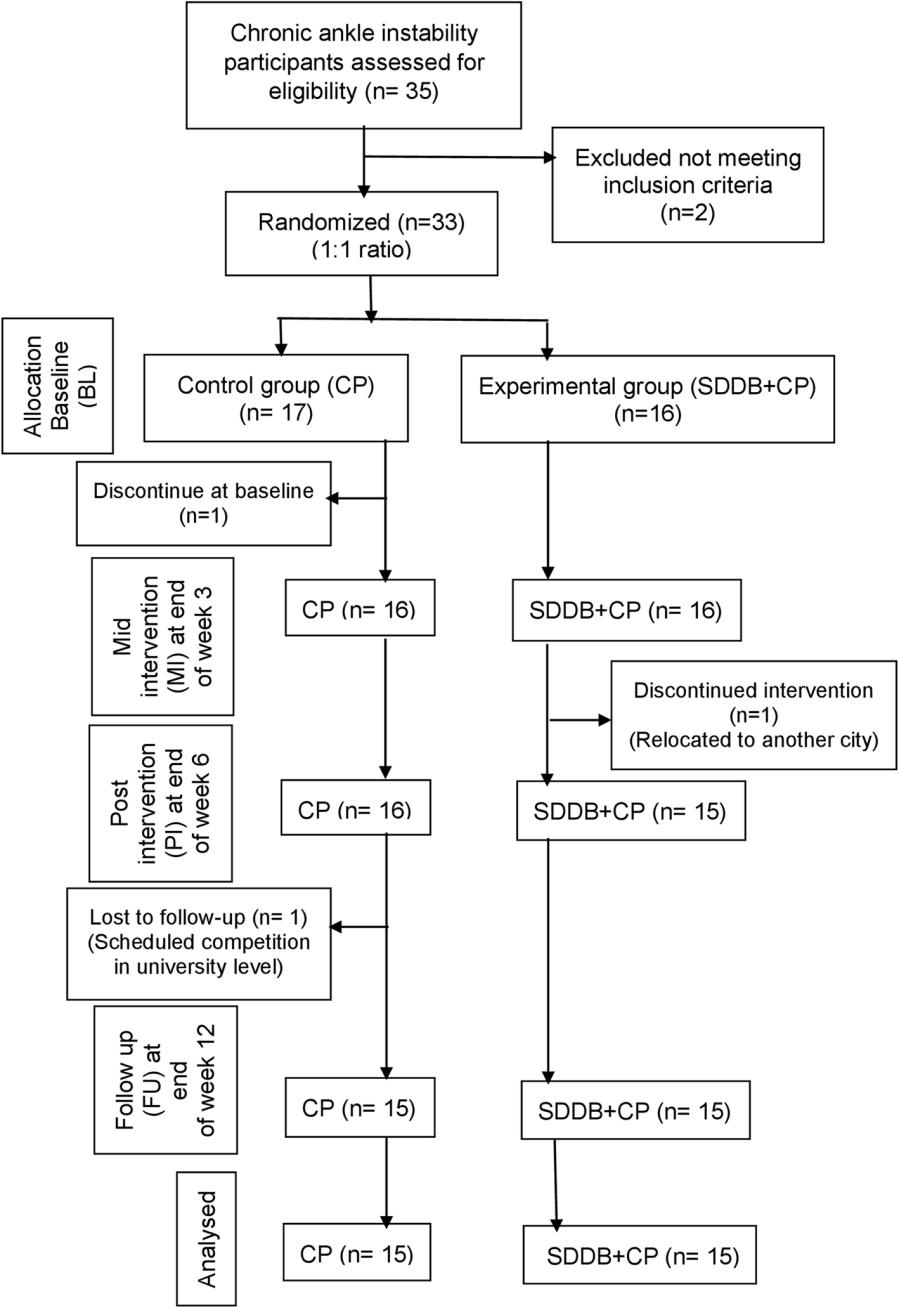
Flow diagram showing the outcome of Chronic ankle instability participants as per CONSORT (2010) guidelines

This study recruited 35 eligible male participants who were diagnosed with repeated ankle sprains and referred by the specialist to the physiotherapy centre during the study enrollment duration. The recruited participants were given unique identification numbers after the computer randomization process by the physiotherapy centre maintenance officer and allocated into two groups as blinded. The study's preliminary assessment and conventional physiotherapy management were carried out by a clinical physiotherapist who specializes in musculoskeletal conditions. The participants in the control group (*n* = 17) received CP, while the participants in the experimental group (*n* = 16) received combined interventions of SDDB and CP. The inclusion criteria of this study were CAIT scores of ≤ 27, age ranging from 18 to 25, male participants, previous history of ankle sprain, and referral by the medical specialist to the physiotherapy centre for ankle instability rehabilitation. The participants with a prior history of surgeries, prescribed with any pain-relieving medicine, previous participation in deep breathing or meditation, both ankle instability and acute injuries to the musculoskeletal structures (muscles, bones and joints) in the lower limbs were all excluded. During the intervention up to week 6, the participants were not allowed to have sports activity for preventing the recurrence of injury. Three participants who failed to attend the follow-up rehabilitation sessions including two from control group (*n* = 2) and one from experimental group (*n* = 1) were excluded from this study (Fig. 1).

Upon arrival at the physiotherapy centre, the participants were administered the questionnaire for the VAS, CAIT, MAAS and OHQ. The parameters of the present study were assessed at different intervals from baseline (BL) where the measurements made before the participants were exposed to the intervention, mid-intervention (MI) at the end of week 3 following the early functional rehabilitation phase, post-intervention (PI) at the end of week 6 following the intermediate functional and advanced rehabilitation phase before returning to the game, and follow-up (FU) at the end of week 12 to monitor the post-intervention effects while they were involved in sports activities.

### Measures

#### Visual analog scale

For pain assessment, VAS was used to record the participants self-reported pain intensity on a 10 cm line to point out the pain level. The point at 0 cm represents "no pain" and 10 cm represents "worst pain". The same scale was used from baseline until follow-up sessions to monitor the progress of the pain. The VAS pain score is a reliable and valid scale with intraclass correlation coefficient (ICC) = 0.76 to 0.84 to assess chronic musculoskeletal pain [44].

#### Cumberland ankle instability tool

The CAIT tool, which has a 9-item Questionnaire, was used to find the chronic ankle disability score among participants and to recruit them into the study if the score was ≤ 27 over 30 [16]. The same tool was used to monitor the participants' progress until the follow-up session with scores ranging from 0 to 30. A higher score represents the stability of an ankle. CAIT has been reported to be a reliable and valid tool with an ICC = 0.83 in testing ankle stability [16].

#### Mindful attention awareness scale

The participants’ mental health and mindfulness state were assessed by MAAS, involving a 15-item questionnaire during the baseline, mid-intervention, post-intervention, and follow-up assessments. The changes in the mental adaptation of participants before and after the intervention were evaluated based on the MAAS score, which was obtained from the Likert scale rating from 1 (almost always) to 6 (almost never). The internal consistency of MAAS among adults has been reported with ICC = 0.81to 0.86 [45].

#### Oxford happiness questionnaire

Besides mindfulness attention, the research looked into the happiness level in pain management from SDDB. The participants happiness levels for both groups were assessed using the Oxford happiness questionnaire OHQ, which consists of a 29-item questionnaire. The OHQ score was recorded on a 6 point Likert scale from 1 (strongly disagree) to 6 (strongly agree) and the OHQ has been established to be a reliable tool with an ICC = 0.87–0.92 for assessing the happiness level among human participants [22, 28, 46].

### Intervention

#### Short-duration deep breathing

The participants in the experimental group were given a 3-min SDDB with visual guidance on deep breathing with 6 deep breaths per minute along with the CP protocol 5 times a week for 6 weeks. The chronic ankle instability participants in both groups underwent CP 5-times a week for 6 weeks [47]. The participants in the experimental group were assisted to access the Google Play store (https://play.google.com/store/apps/details?id=com.vamdb.asus.happyproject) to install VAMDB (Video-Aided Mindful Deep Breathing) on their smartphone. Following each session of CP at the physiotherapy centre, the participants in the experimental group (EG) were guided to perform SDDB for 3 min at the physiotherapy centre. Furthermore, the participants in the EG were instructed to continue the same practice of 3 min of SDDB as a home exercise in the evening. In total, they were exposed to SDDB two times a day. The participants in the CP were instructed to continue home exercise in the evening as prescribed. Besides, the participants were reminded of the practice through the text messages, and their practice schedule was monitored with a reply message from the participants on a daily basis.

**Fig. 2:**
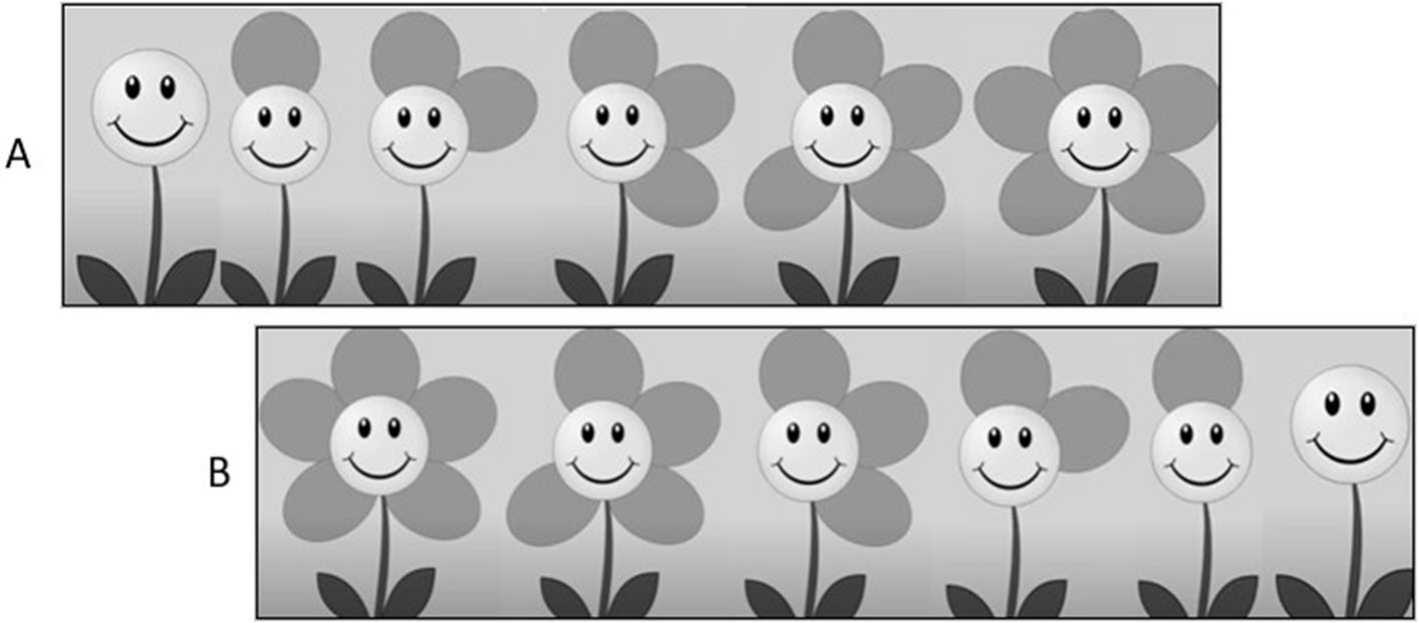
**A** Breath-in and **B**. breath-out**—**Screenshots from the 3-min short duration mindfulness deep breathing exercise [48]

The SDDB involves a video demonstration, which displays a flower with a smiley face in the center surrounded by petals, visually guiding the participants to deep breathing. The petals appear and disappear in a timely manner, where the participants were instructed to focus on the smiley face of the flower and asked to breathe in during the appearance of the petals and then breathe out during the disappearance of the petals to standardize the participants breathing pattern (Fig. 2A and B) [41, 48]. Meanwhile, control group participants had not been introduced to VAMDB tool.

### Statistical analysis

The sample size was determined to be 36 participants (*n* = 36) with G*power, a medium effect size of 0.25, an alpha error probability of 0.05, and a power of 95% [49, 50]. The results of outcome measures at baseline, mid-intervention, post-intervention, and follow-up sessions were analysed using SPSS version 22.0 (SPSS, Inc). The descriptive analysis was applied to report the demographic characteristics of CAI participants. The nonexistence of a significant difference in the baseline measures between the control group (CG) and experimental group (EG) was confirmed by an independent *t*-test (*p* > 0.05). The normal distribution of the data was determined using the Shapiro–Wilk test. Two-way repeated-measures analysis of variance (ANOVA) was performed to test the significant difference on the intervention effects between the time and group*time intervals (baseline, mid-intervention, post-intervention, and follow-up) and also between the groups (EG and CG). Post–hoc analysis Bonferroni conducted if significant effects within the group*time interaction and between the group intervals were identified. The* p* value for time interaction was set as 0.0125 (0.05/4). If the sphericity condition was violated (*p*-value < 0.05), the Greenhouse–Geisser estimated corrections were applied. The effect size of the outcome measures within and between the groups the Cohen’s *d* were calculated (0.20 = small, 0.50 = medium, and 0.80 = large) and interpreted [51].

## Results

### Demographic and baseline characteristics

The study reported the results of 30 CAI participants who completed the ankle rehabilitation protocol and their demographic and baseline characteristics in Table 1. The demographic details of participants showed that 6 (20%) were Malaysian Malay, 7 (23.33%) were Malaysian Indians, and 17 (56.67%) were Malaysian Chinese. The study population included young adults from various sports activities; 7 (23.3%) from football, 8 (26.7%) from basketball, 5 (16.7%) from badminton, 2 (6.7%) from frisbee, 4 (13.3%) from martial arts, and 4 (13.3%) from futsal. Further, the majority of the recruited participants (83.35%) were reported to be right-leg dominant in the sports they played. Remarkably, it is evident from the demographic data that the injury occurred on the dominant side of the youth athletes, as the right ankle injury was reported among right-leg dominant. Furthermore, the baseline data of CAI participants for pain, balance, attention, and happiness, showed no significant difference (*p* > 0.05) for the independent *t*-test between the control and experimental groups, as reported in Table 1.

**Table 1:** Demographic and baseline characteristics of study participants

**Variable**	**Control group (CG) (*n* = 17)**	**Experimental group (EG) (*n* = 16)**	***t***	***p*-value**
Age (years)	21.65 (1.84)	21.75 (2.20)	0.14	0.88
Age range (years)	18–24	18–25		-
**Dominant side (leg)**				
Right, n (%)	15 (88.2%)	12 (80%)		-
Left, n (%)	2 (11.8%)	3 (20%)		-
**Affected side (leg)**				
Right, n (%)	13 (76.5%)	9 (56.3%)		-
Left, n (%)	4 (23.5%)	7 (43.8%)		-
**Outcome Measures**				
VAS (pain intensity) intensity)	4.35(1.32)	4.31(1.40)	-0.08	0.93
CAIT	20.18(2.24)	19.75(4.02)	-0.37	0.71
MAAS	61.47(11.46)	61.63(12.09)	0.03	0.97
OHQ	4.05(0.51)	4.29(0.57)	1.31	0.20

Data are given as mean & SD

*CAIT* Cumberland ankle instability tool, *VAS* Visual analogue scale for pain, *MAAS* Mindful attention awareness scores, *OHQ* Oxford Happiness Questionnaire

### Effects of study intervention

**Fig. 3:**
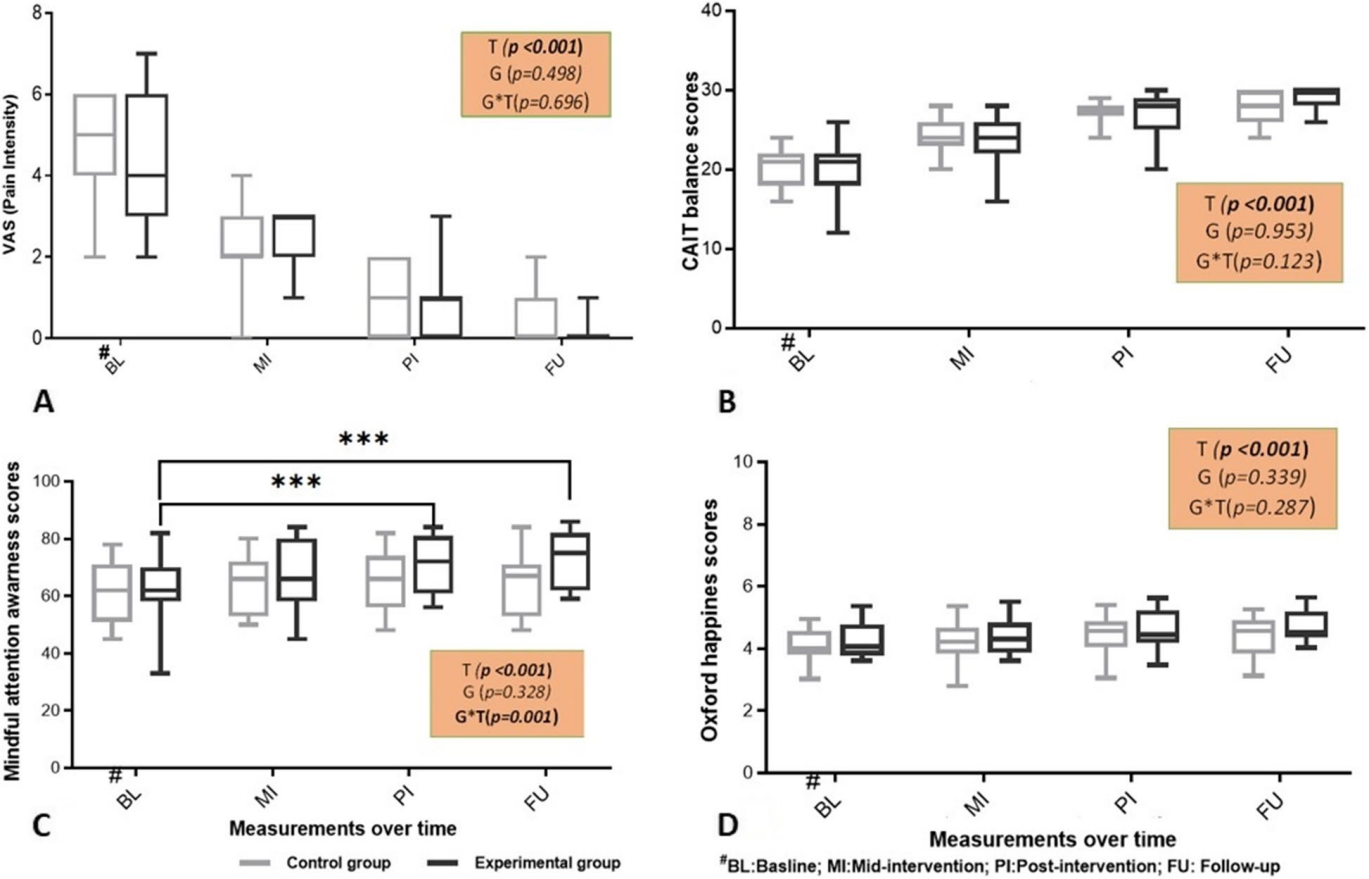
Adjusted mean shown as BL-Baseline at week 1; MI-Mid-intervention at end of week 3; PI- Post-intervention at end of week 6; FU-Follow-up at end of week 12, the outcome measures compared between Control and Experimental group on time points by using two-way repeated measures ANOVA. **A** pain intensity (VAS), **B** dynamic balance (CAIT), **C** Mindful attention (MAAS) and **D** Happiness level (OHQ) for Groups. Values are reported as mean ± SD, *p < 0.05

The present findings in this study were analysed to determine the combined effects of SDDB with CP over CP on improving ankle stability. Figure 3 displays the results of outcome measures of four time points analysed using two-way repeated-measures ANOVA. The reduction in pain level was reported using the VAS scale to assess the beneficial effects of treatments on improving pain among the participants. A 2 × 4 repeated measures result showed participants’ pain intensity level over time intervals was significantly reduced (F (2.264, 63.380) = 105.607, *p* < 0.001, η2 = 0.790). However, time × group interaction (F (2.264, 63.380) = 0.403, p = 0.696, η2 = 0.014), and between the groups (EG vs. CG) reported an insignificant difference (F (1, 28) = 0.470, *p* = 0.498, η2 = 0.017) in participants’ pain intensity. The box plot showed the experimental group participants' mean pain intensity score was reduced from baseline to mid-intervention, baseline to post-intervention, and baseline to follow-up intervals. Similar effects were discovered in the control group since conventional physiotherapy includes pain intervention as part of the standard protocol for CAI. This indicates that participants in both groups responded to the intervention provided, and especially the participants in the experimental group that received SDDB showed better pain relief with a larger effect size (*d* > 0.80) in mid-intervention and post-intervention intervals (Fig. 3A).

An improvement in the dynamic balance due to the interventions was assessed by CAIT tool. A 2 × 4 repeated measures showed a significant improvement in balance scores (F (2.137, 59.833) = 169.981, *p* < 0.001, η^2^ = 0.859) over the period of treatment time. Similar like pain intensity outcome, the balance scores reported no significant differences between the groups (EG vs. CG) (F (1, 28) = 0.004, *p* = 0.953, η2 = 0.000), and the time × group interaction (F (2.137, 59.833) = 2.141, *p* < 0.123, η2 = 0.071). After being exposed to progressive functional rehabilitation, the CAI participants dynamic balance scores improved in both groups. However, the participants’ mean dynamic balance scores were considerably higher in the experimental group compared with the control group from baseline to mid-intervention, baseline to post-intervention, and baseline to follow-up intervals, with a larger effect size (*d* > 0.80) (Fig. 3B). The combined intervention of focused short-duration deep breathing and conventional physiotherapy produced a better improvement in dynamic balance.

MAAS reflects the mental adaptation of the participants towards the attention of the interventions. The attention score reported in this study demonstrates a significant improvement between time interaction F (2.107, 59.008) = 18.298, *p* < 0.001, η2 = 0.395, and time × group F (2.107, 59.008) = 9.054, *p* = 0.001, η^2^ = 0.224. However, no significant difference was reported between the groups (EG vs. CG) F (1, 28) = 0.993, *p* = 0.328. Specifically, the participants' mindful attention scores in the EG showed a significant improvement (*p* < 0.001) from baseline to post-intervention and baseline to follow-up with a larger effect size (*d* > 0.80) as compared with the control group (CG). In particular, the attention score for the follow-up session was observed to be higher in EG (73.06 ± 9.54) compared with in CG (64.93 ± 10.73) as shown in box plot (Fig. 3C). The present findings implies that the implementation of SDDB along with CP resulted in a higher level of mindful attention than CP. Likewise, the Oxford happiness score showed a significant improvement in happiness level over time intervals, F (2.251, 63.028) = 22.599, *p* < 0.001, η^2^ = 0.447. However, there was no statistical difference in happiness scores between the two groups (F (1, 28) = 0.735, *p* = 0.399, η2 = 0.026) and time × group interaction (F (2.251, 63.028) = 1.279, *p* = 0.287, η2 = 0.044) (Fig. 3D). Whereas, the mean values were observed to be increased in happiness score from baseline to mid-intervention, baseline to post-intervention, and baseline to follow-up in the experimental group. Similar effects were revealed among participants in the control group. In accordance with the improvement in pain, the improvement in happiness score was observed in both the experimental and control groups. Remarkably, the happiness mean score in the follow-up session was comparatively higher in the EG. The results of the Oxford happiness scale show that the SDDB has brought a better outcome in the experimental group by increasing the happiness level compared with control group participants. It is inferred from the findings that SDDB may play a major function in maintaining the happy feelings and mindful attention of the individual, which in turn may result in the prevention of the recurrence of ankle injuries.

## Discussion

The present study examined the effects of 6-week conventional physiotherapy (CP) along with 3-min short-duration deep breathing (SDDB) through a smartphone application for participants with chronic ankle instability (CAI). It was aimed at guiding the participants through the same breathing rhythms to enhance the accuracy of breathing training patterns [39, 43, 52]. As we expected, the combined intervention (SDDB + CP) has been found to deliver a substantial improvement in reducing the pain intensity and improving the balance, mindfulness attention, and happiness levels of chronic ankle instability (CAI) participants. Similarly, a 6-week CP in the control group (CG) resulted in an improved pain intensity, dynamic balance, and happiness level. However, no considerable improvement in mindfulness attention was observed in CG. Based on the findings, the study illustrates the benefits of SDDB + CP in improving participants' mindfulness attention towards preventing CAI.

Numerous studies in health care have shown that deep breathing is an effective coping strategy for pain [31, 53] and well-being [41, 54, 55]. In previous studies, the effect of short-duration breathing was mostly applied to healthy participants [37, 41]. The SDDB with a smartphone was able to help participants stay focused on their breath, which relieves the mental strain in accordance with polyvagal theory [29], and hence was able to prevent the progression of pain and disability [56]. A report showed that the pain tolerance levels of injured athletes were reduced by following an 8-week period with 90 min of formal mindfulness-based breathing practise [57]. However, the present study with 3-min SDDB for 6 weeks was found to be effective in reducing pain with a notable decrease in pain intensity among the experimental group (EG) participants who underwent the combined interventions when compared with CP. Similarly, the previous work among chronic pain participants who underwent formal mindfulness training showed no significant difference among who received CP [57, 58]. The findings confirm that CP combined with breathing-based interventions for a short period of time produces better pain relief.

A previous report findings from a clinical trial among healthy participants who trained for 8 weeks in various breathing techniques were able to improve the participants' balance scores, giving confidence that the breathing exercise is capable of improving the balance [59]. However, the study reported the improvement in static single leg balance scores of healthy participants [59], whereas our study reported the improvement in balance mean score among CAI participants. An increase in balance mean score was found to have a larger effect on SDDB from baseline to follow-up, indicating the beneficial effects of ankle rehabilitation in improving the dynamic balance of CAI participants in EG. It is evidenced from the literatures that the breathing has the ability to improve the diaphragmatic core muscle strength and thus can improve the participants balance by altering the cortical activation of the brain, which is commonly altered in participants with CAI [9, 59]. In accordance with the improvement in pain and balance, the results also reported a significant (*p* < 0.05) increase in attention score due to SDDB. In line with our study findings [60], reported an improved attention using attention score on student learning abilities following a 8-week formal mindfulness intervention. On the other hand, the same study reported no improvement in attention level by 3 weeks of device guided slow breathing for 15 min. Similarly, the present study attention score showed no improvement in mindfulness attention following SDDB at week 3 during mid-intervention assessment, whereas it showed an improvement in attention score at week 6 in post-intervention and at week 12 in follow-up. This outcome has led to the speculation concept that the longer the intervention duration, in practise, increases the attention level of the participants. In addition to the improvement in attention level, the happiness level reported in this study is also found to be increased due to SDDB until follow-up. This finding is supported by the earlier studies which had reported a positive relationship of mindfulness with happiness and thus improved coping skills, self-esteem, and decreased level of stress [21, 23, 38, 61]. Also, numerous studies have indicated that the focused breathing and mindfulness techniques benefit the improvement in pain tolerance, increased attention, and happiness level [19, 21, 23, 60, 62, 63]. The implications of these study findings indicate that breathing techniques influence the physiological response of individuals by improving attention and happiness, which recommends the biopsychosocial approach in chronic injury rehabilitation. However, the present study have few limitations by not comparing male and female participants by selecting a specific sport participant from team or individual sports [64].

For limitation, this pilot study could be improved by including a healthy population without injury as an active control group, as this study only worked on injured CAI participants with and without 3-min video guided SDDB, and both groups underwent conventional physiotherapy. Further studies are required to compare the physiological effects of different durations of deep breathing (5, 7, and 9-min) [52] on various chronic musculoskeletal conditions for well-being. Furthermore, psychological parameters of participants, such as salivary cortisol levels, memory tests, anxiety, stress, and depression inventories, could be investigated to assess improvements in participants' psychological states as the benefits of interventions.

## Conclusion

The findings of the present pilot study demonstrated that the combined interventions of SDDB with CP for a period of 6-weeks caused an improvement in pain, balance, happiness, and attention among young adults with CAI. The progressive effects perceived by the short-duration deep breathing using smartphone application among young adults as a tool in pain management, bring an additional benefit along with the existing CP to improve the well-being of injured athletes during rehabilitation.

### Supplementary Information

**Additional file 1. **

## Data Availability

The datasets used and/or analyzed during the current study are available from the corresponding author.

## Abbreviations

ANOVA: Analysis of Variance
CAI: Chronic Ankle Instability
CG: Control Group
CP: Conventional Physiotherapy
EG: Experimental Group
MAAS: Mindful Attention Awareness Scores
OHQ: Oxford Happiness Questionnaire
SDDB: Short-Duration Deep Breathing
VAS: Visual Analogue Scale
